# Sequential Depletion and Acquisition of Proteins during Golgi Stack Disassembly and Reformation

**DOI:** 10.1111/j.1600-0854.2010.01106.x

**Published:** 2010-08-18

**Authors:** Jennifer Schoberer, John Runions, Herta Steinkellner, Richard Strasser, Chris Hawes, Anne Osterrieder

**Affiliations:** 1 Department of Applied Genetics and Cell Biology, University of Natural Resources and Applied Life SciencesVienna, Muthgasse 18, 1190 Vienna, Austria; 2 School of Life Sciences, Oxford Brookes University, Headington CampusGipsy Lane, Oxford OX3 0BP, UK

**Keywords:** Brefeldin A, golgins, Golgi apparatus, *N*-glycan processing enzymes, Sar1p GTPase

## Abstract

Herein, we report the stepwise transport of multiple plant Golgi membrane markers during disassembly of the Golgi apparatus in tobacco leaf epidermal cells in response to the induced expression of the GTP-locked Sar1p or Brefeldin A (BFA), and reassembly on BFA washout. The distribution of fluorescent Golgi-resident *N*-glycan processing enzymes and matrix proteins (golgins) with specific *cis*–*trans*-Golgi sub-locations was followed by confocal microscopy during disassembly and reassembly. The first event during Golgi disassembly was the loss of *trans*-Golgi enzymes and golgins from Golgi membranes, followed by a sequential redistribution of medial and *cis*-Golgi enzymes into the endoplasmic reticulum (ER), whilst golgins were relocated to the ER or cytoplasm. This event was confirmed by fractionation and immuno-blotting. The sequential redistribution of Golgi components in a *trans–cis* sequence may highlight a novel retrograde trafficking pathway between the *trans*-Golgi and the ER in plants. Release of Golgi markers from the ER upon BFA washout occurred in the opposite sequence, with *cis*-matrix proteins labelling Golgi-like structures before *cis*/medial enzymes. *Trans*-enzyme location was preceded by *trans*-matrix proteins being recruited back to Golgi membranes. Our results show that Golgi disassembly and reassembly occur in a highly ordered fashion in plants.

The plant Golgi apparatus has a unique architecture and is organized as polarized stacks of flattened cisternae that exhibit a structural and functional *cis*-to-*trans* polarity (1,2). Despite their morphology, Golgi membranes are highly dynamic and undergo rapid disassembly and reassembly in response to various perturbations in membrane trafficking pathways operating between the Golgi apparatus and ER (3).

Golgi bodies are intimately associated with the ER in many plant tissues and have been shown to move with the motile surface of the ER and ER exit sites (ERES), forming a ‘mobile secretory unit’ in *Nicotiana tabacum* (tobacco) leaf epidermal cells (1,4,5). It is not known how Golgi stacks are formed and how they maintain their structure and connection with the ER during movement, but possibly Golgi-localized coiled-coil proteins, or golgins, might play an important role. In animals, golgins are involved in Golgi stack assembly, integrity and tethering events (6–8). A number of putative homologues have been identified in plants (1,9–13) and a model for *de novo* formation of plant Golgi stacks from ERES involving golgins has recently been proposed (1).

In the Golgi apparatus, resident glycosyltransferases and glycosidases are organized across the stack into an assembly line for the sequential processing of protein- or lipid-associated glycans and hence biochemically define the *cis*-, medial- and *trans*-Golgi cisternae and the *trans*-Golgi network (TGN) (14–16). Although considered ‘resident’, many, if not all, Golgi proteins continuously recycle both within the Golgi apparatus itself and between the Golgi apparatus and the ER (17–19). The steady-state distribution of Golgi processing enzymes is thought to be, in part, the result of this recycling. It could occur either through regular coat protein I (COPI)-dependent retrograde vesicular transport, a COPI-independent mechanism involving tubules, direct transport to the ER, or some combination of these possibilities (19,20). Evidence for the cycling of Golgi residents, for example, comes from fluorescence recovery after photobleaching (FRAP) experiments that demonstrated exchange of green fluorescent protein (GFP)-tagged Golgi proteins between Golgi and ER pools (21–23). More evidence in favour of recycling comes from the over-expression of a GTP-locked Sar1p, the small GTPase which initiates COPII coat formation at ERES, leading to a block in ER exit, as well as the addition of Brefeldin A (BFA), which blocks assembly of COPI vesicles (24). Both perturbations induce disassembly of Golgi cisternae and redistribution of Golgi membrane markers into the nearby ER network (4,23,25–30), an event similar to that reported for mammalian cells (21,31–35). It is well documented that the washout of BFA from treated plant cells (27,28,36) triggers reformation of the Golgi apparatus, even when both actin filaments and microtubules are depolymerized and when protein synthesis is inhibited by cycloheximide. This behaviour is consistent with the idea that Golgi membranes are in dynamic equilibrium with the ER and have the capacity to form *de novo* by dynamic self-organization of Golgi components as they exit the ER.

In a recent study of Golgi membrane dynamics in tobacco leaf epidermal cells we showed that the redistribution of the two *cis*/medial-Golgi matrix proteins AtCASP and GC1 (Golgin Candidate 1, an *Arabidopsis* golgin-84 isoform) to the ER and cytoplasm, respectively, was preceded by the relocation of the *trans*-Golgi membrane marker ST (rat sialyltransferase) after BFA treatment and after induction of Sar1-GTP expression (30). This event indicated differences in the distributional persistence of a putative Golgi matrix and a membrane-bound Golgi enzyme.

Here we have exploited previously characterized Golgi-resident *N*-glycan processing enzymes and matrix proteins tagged with fluorescent proteins as markers for different Golgi sub-compartments to examine the dynamics of the Golgi apparatus *in vivo* in response to experimentally induced perturbations. By confocal microscopy, we sequentially monitored the marker distribution in tobacco leaves during Golgi disassembly triggered by BFA or Sar1-GTP (30), and reassembly upon BFA washout. Our results show that in plants the deconstruction and *de novo* reformation of Golgi stacks are highly ordered processes that occur in a directional manner.

## Results

### N-glycan processing enzymes define distinct regions of the Golgi apparatus in plants

To study the fate of individual cisternae (or sub-compartments) during Golgi disassembly and reassembly we used fluorescent protein-tagged integral Golgi-resident *N*-glycan processing enzymes, which are differentially localized in sequential Golgi cisternae according to their position in the biosynthetic pathway. In a previous study, it has been shown by coexpression with the established *trans*-Golgi membrane marker ST-monomeric red fluorescent protein (ST-mRFP) that it was possible to identify by confocal microscopy whether fluorescent glycosyltransferases and glycosidases locate to early- or late-Golgi sub-compartments (37).

**Figure 1:**
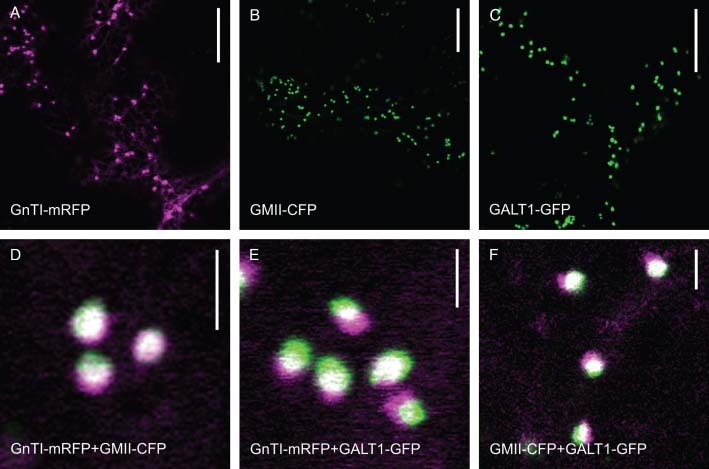
**GnTI-mRFP, GMII-CFP and GALT1-GFP show distinct intra-Golgi distributions.** Confocal images showing GnTI-mRFP, GMII-CFP and GALT1-GFP transiently expressed alone (A–C) or in pairs (D–F) in wild-type tobacco leaf epidermal cells. Double-colour images (D–F) display highly magnified Golgi stacks double-labelled by (D) GnTI-mRFP (magenta) and GMII-CFP (green), (E) GnTI-mRFP (magenta) and GALT1-GFP (green) or (F) GMII-CFP (magenta) and GALT1-GFP (green). Note the shift of the overlapping signals with the central region being white and the non-overlapping regions being green and magenta. Scale bars = 20 µm in (A–C) and 2 µm in (D–F).

We re-examined the intra-Golgi distribution of the three *N*-glycan processing enzymes GnTI-mRFP, GMII-cyan fluorescent protein (GMII-CFP) and GALT1-GFP by confocal microscopy after transient expression of their signal anchor sequences in tobacco leaf epidermal cells (Table 1). Consistent with previous studies (37–39), we observed GnTI-mRFP in the Golgi apparatus and, to a much lesser extent, in the ER (Figure 1A), while GMII-CFP and GALT1-GFP located exclusively to Golgi bodies (Figure 1B,C). An expression of the three Golgi markers in pairs revealed that many labelled Golgi stacks clearly appeared tricolored (Figure 1D–F). These differences in labelling pattern which were also observed by coexpression with ST-mRFP or ST-GFP (data not shown) likely reflect the location of the studied constructs to different sets of Golgi cisternae. The most significant difference was detected between the *cis*/medial-located GnTI-mRFP and the *trans*-Golgi enzyme GALT1-GFP (Figure 1E). GnTI-mRFP and GMII-CFP possibly reside in adjacent cisternae of a stack. However, colocalization signals always overlapped, indicating that Golgi enzymes are not restricted to one or two cisternae and are better thought of as markers for a broader region of the Golgi apparatus rather than for individual cisternae.

**Table 1:** Properties of Golgi marker proteins used

Protein	Protein type	Cisternal location	Tag	Reference
Glycosyltransferases/glycosidases				
GnTI^a^	Type II membrane	*cis/*medial	mRFP GFPglyc	(39)
GMII^b^	Type II membrane	medial	CFP GFP	(39)
GALT1^c^	Type II membrane	*trans*	GFP	(38)
ST^d^	Type II membrane	*trans*	GFP mRFP	(28)(11)
Golgins^e^				
AtCASP	Type II membrane	*cis/*medial	GFP mRFP	(12)(40)
GC1	Type II membrane	*cis/*medial	GFP	(12)
GC5	Peripheral	*trans*	GFP	(12)
AtGRIP	Peripheral	*trans*/TGN	GFP	(10,12)

a GnTI: the first 77 N-terminal amino acids (CTS region) of the tobacco β1,2-*N*-acetylglucosaminyltransferase I.

b GMII: 32 or 52 N-terminal amino acids (C_10_T or C_10_TS region) of the *Arabidopsis* Golgi α-mannosidase II.

c GALT1: the first 60 N-terminal amino acids (CTS region) of the *Arabidopsis*β1,3-galactosyltransferase.

d ST: the first 52 N-terminal amino acids (CTS region) of the rat α2,6-sialyltransferase.

e Golgins: full-length *Arabidopsis* Golgi matrix proteins.

### Golgi disassembly is accompanied by the sequential redistribution of Golgi markers to the ER in a *trans*-to-cis order

We examined the distribution of GnTI-mRFP, GMII-CFP and GALT1-GFP at different time-points during Golgi breakdown in response to BFA treatment of wild-type cells or induction of Sar1-GTP expression in transgenic tobacco plants. Even though in leaves the primary effects of both methods, which are the disruption of Golgi membranes and the redistribution of Golgi membrane markers into the ER, appear to be similar, both approaches differ in their mode of action. BFA has a relatively rapid effect on organelles and has multiple targets within the cell, whilst the inducible Sar1-GTP system constitutes a more controlled approach at the genetic level by targeting only one specific step within the secretory pathway (30).

**Figure 2:**
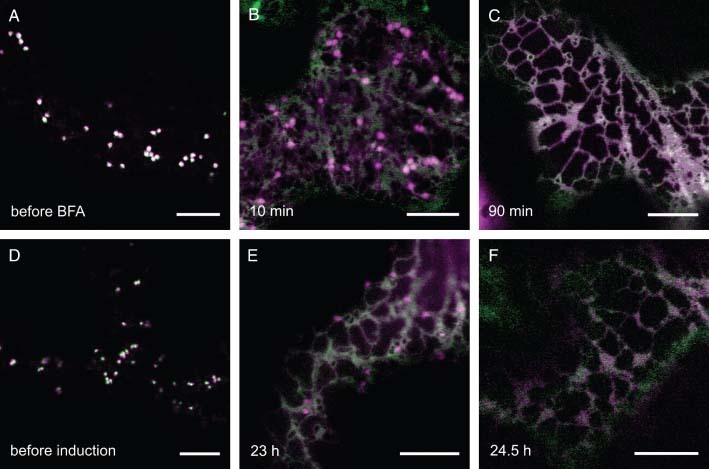
**GALT1-GFP is redistributed into the ER before GnTI-mRFP during Golgi disassembly.** Time–course of the effects of BFA and Sar1-GTP expression, respectively, on the Golgi markers GnTI-mRFP (magenta) and GALT1-GFP (green). The expression of markers was either checked before and after treatment of wild-type tobacco leaves with 100 µg/mL BFA over a time-period of 2–3 h (A–C) or before and after induction of stable inducible tobacco plants with a 20 µg/mL dexamethasone solution over a time-period of 18–25 h (D–F). Double-colour images (a merge of green and magenta channels) were obtained by confocal microscopy at different time-points of each experiment. Scale bars = 10 µm.

When GnTI-mRFP and GALT1-GFP were coexpressed in wild-type tobacco leaf epidermal cells, they were seen to overlap in their Golgi distribution (Figure 2A). Unexpectedly, only minutes after BFA addition, the first event observed was a loss of the *trans*-Golgi enzyme GALT1-GFP from most Golgi stacks and a build-up of GALT1-GFP fluorescence in the ER, whilst the GnTI-mRFP signal continued to label Golgi bodies (Figure 2B). By 90 min, fluorescence of both enzymes was found in the ER (Figure 2C). This finding was independent of the fluorescent protein-tag used, as following BFA treatment of ST-mRFP and GnTI-GFPglyc (Table 1) the ST-mRFP signal was observed in the ER before GnTI-GFPglyc (data not shown).

**Figure 3:**
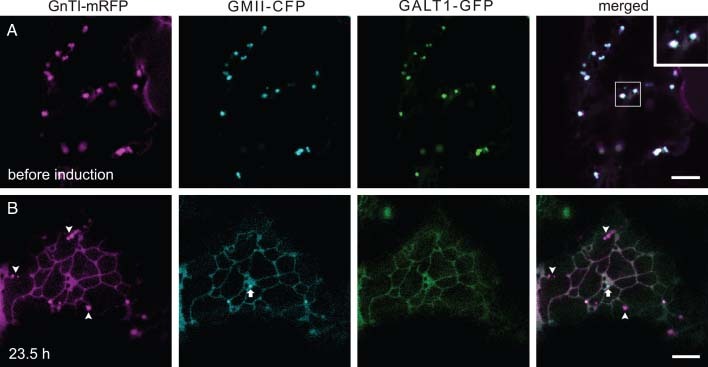
**Golgi disassembly occurs in a *trans*-to-*cis* direction.** Confocal images showing GnTI-mRFP (magenta), GMII-CFP (cyan) and GALT1-GFP (green) transiently expressed together in leaves of stable Sar1-GTP-inducible tobacco plants before (A) and 23.5 h (B) after treatment with dexamethasone (20 µg/mL). The inset (A) shows a magnification of the shift of overlapping signals within triple-labelled Golgi stacks. The arrow (B) indicates a Golgi stack solely labelled by GMII-CFP, whereas arrowheads indicate stacks labelled only by GnTI-mRFP. Scale bars = 5 µm.

The same sequence of events was observed when GnTI-mRFP and GALT1-GFP were expressed in stable Sar1-GTP-inducible tobacco plants and leaves were treated with dexamethasone (Figure 2D-F). At 23 h after treatment, the redistribution of GnTI-mRFP to the ER clearly lagged behind that of GALT1-GFP (Figure 2E). Colocalization in the ER of both markers was complete after 24.5 h (Figure 2F). Double-expression experiments of GnTI-mRFP/GMII-CFP and GMII-CFP/GALT1-GFP (Figure S1), respectively, using both the BFA and the inducible Sar1-GTP approach confirmed that Golgi markers are redistributed into the ER in a stepwise fashion. Treatment with cycloheximide, a protein synthesis inhibitor, alongside BFA had no effect on ER fluorescence, indicating that the effect was due to Golgi proteins in the ER rather than *de novo* protein synthesis (Figure S2). We also followed the distribution of all three enzymes in parallel on induction of Sar1-GTP expression which permitted a more controlled deconstruction of Golgi stacks for tracing differential redistribution patterns. Before induction, all three proteins located to the Golgi apparatus (Figure 3A). The first event on Golgi disassembly was the loss of GALT1-GFP, which was followed by the sequential redistribution of GMII-CFP and then GnTI-mRFP into the ER (Figure 3B). Redistribution occurred exactly in a *trans*-to-*cis* direction, which corresponds to the results described above.

**Figure 4:**
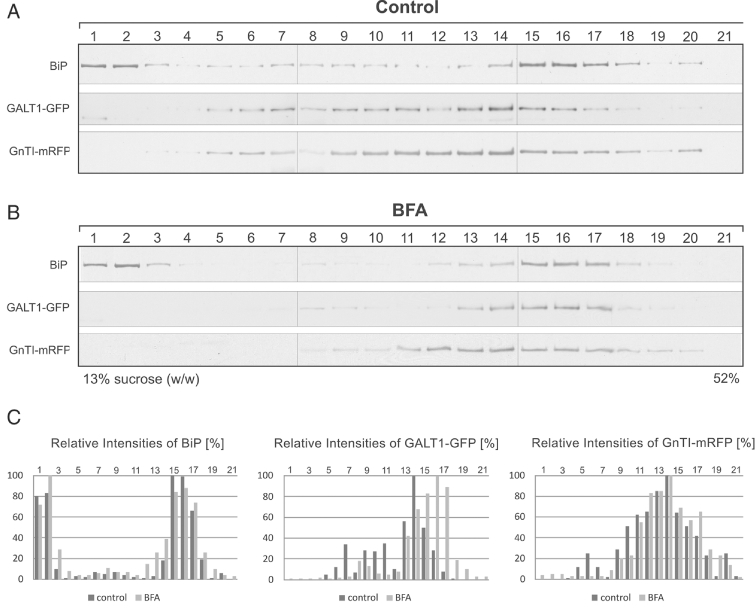
**Subcellular fractionation analysis of the effect of BFA on GnTI-mRFP and GALT1-GFP expressing cells.***N. benthamiana* leaf epidermal cells transiently coexpressing GnTI-mRFP and GALT1-GFP were treated with BFA (75 µg/mL) for 45 min. Microsomes were obtained from mock-treated (control, A) and BFA-treated (BFA, B) leaf tissue and separated on a discontinuous sucrose density gradient. Each of the 21 fractions was subjected to immunoblot analysis with antibodies against BiP (ER marker), GFP (GALT1-GFP) and mRFP (GnTI-mRFP). Fractions were numbered from top (fraction 1 in the first lane on the left) to bottom (fraction 21 in the last lane on the right) of the gradient. In order to compare the migration behaviour of each protein after control and BFA treatments, respectively, the intensity of bands on each immunoblot was quantified and expressed as relative intensities (in %, C).

Our findings were confirmed biochemically by subcellular fractionation of microsomes from wild-type *Nicotiana benthamiana* leaves transiently expressing GnTI-mRFP and GALT1-GFP, after a 45-min BFA treatment (Figure 4). In control cells (no BFA added), the ER-resident chaperone BiP (41), a well-established ER marker used for subcellular fractionation/localization studies in plants (42–44), distributed in heavier membrane fractions with peaks in fractions 15 and 16, but was also detected in much lighter fractions at the top of the gradient (fractions 1 and 2), which is due to leakage of some soluble contents from the ER lumen (Figure 4A,C). The *trans*-Golgi marker GALT1-GFP clearly peaked in fraction 14, whilst the *cis/*medial-Golgi marker GnTI-mRFP showed a broader distribution (fractions 11–15) with a peak in fraction 14, the same as GALT1-GFP. A co-migration of either of the two Golgi markers with other organelle fractions was not detected. In general, the distribution of ER and Golgi membrane fractions slightly overlapped because it is technically challenging to separate the membranes of these two organelles completely without any contamination (45). After BFA incubation, BiP distribution was similar to that in control cells (Figure 4B,C). GnTI-mRFP distribution was almost unchanged (peak in fraction 14), except for a small amount of GnTI-mRFP increasing in ER membrane fractions (fractions 15–17), indicating relocation of a small portion to the ER. Interestingly, after BFA treatment the distribution of GALT1-GFP clearly shifted to heavier membrane fractions with a peak in fraction 16, the same as the ER marker BiP. This shift in protein towards heavier fractions on BFA treatment can also be observed in silver-stained protein gels (data not shown). These results support the observations from confocal multi-marker analyses in favour of a *trans*-first Golgi protein cycling to the ER.

### Golgi markers are released from an ER exit block at different rates

**Figure 5:**
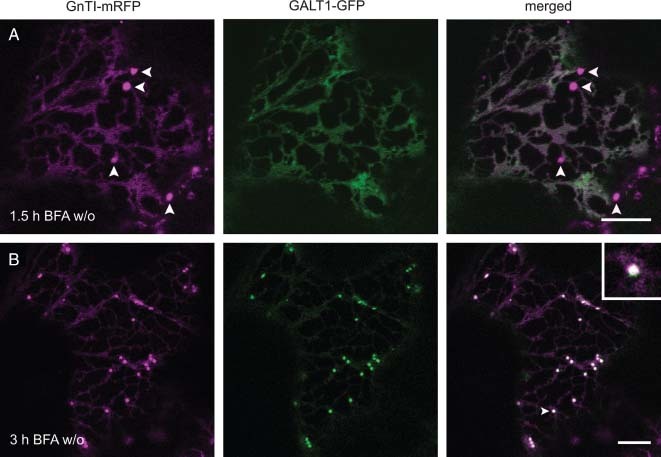
**GnTI-mRFP is found at reforming Golgi bodies before GALT1-GFP after BFA washout.** Confocal images showing wild-type tobacco leaf epidermal cells coexpressing GnTI-mRFP (magenta) and GALT1-GFP (green) 1.5 h (A) and 3 h (B) after BFA washout (w/o). Before removal of BFA, Golgi proteins were forced back into the ER by a 2-h BFA treatment (100 µg/mL). Scale bars = 10 µm.

To study the dynamics of GnTI-mRFP and GALT1-GFP during Golgi reassembly we forced both markers back into the ER with BFA before testing for reassembly (Figure 5). GALT1-GFP was seen only in the ER 1.5 h after BFA removal, whereas GnTI-mRFP fluorescence labelled the ER and additionally concentrated in small mobile punctate structures resembling Golgi bodies (Figure 5A, arrowheads). After 3 h, both Golgi proteins ultimately appeared to colocalize at reformed Golgi stacks (Figure 5B, arrowhead and inset). At that time-point, residual ER labelling by both constructs was still visible. This result suggests a stepwise transport of Golgi membrane markers from the ER to the Golgi in a *cis*-to-*trans* direction.

### Golgi processing enzymes with distinct intra-Golgi locations cycle in and out of Golgi bodies at similar rates

**Figure 6:**
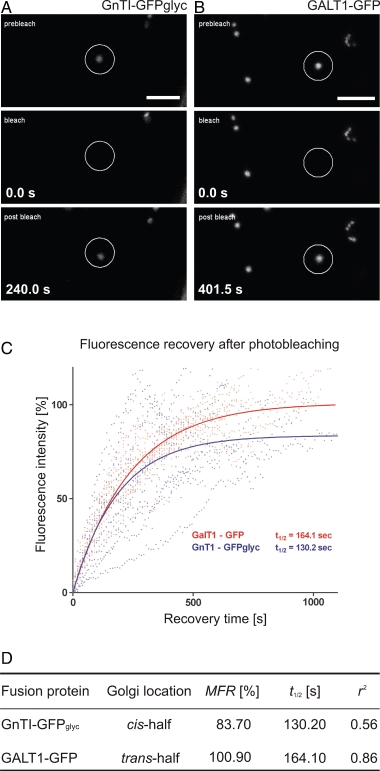
**GnTI-GFPglyc and GALT1-GFP have similar dynamic behaviours.** A and B) Confocal images showing a time series of the fluorescence recovery in photobleached Golgi stacks (circles) in tobacco leaf epidermal cells transiently expressing either GnTI-GFPglyc (A) or GALT1-GFP (B). Before bleaching, cells were treated for 1 h with latrunculin B (25 µm). Time is expressed in seconds (s) at the bottom left of frames. Scale bars = 5 µm. (C) Fluorescence recovery graph: measured, normalized and fitted FRAP plotted against time. Values for maximum fluorescence recovery (MFR), *t*_1/2_ (fluorescence half-time of recovery) and curve fit *r*^2^ were derived from these data and are summarized in (D).

To test whether GnTI and GALT1 have different Golgi residency times because of their distinct intra-Golgi distributions, we selectively photobleached individual Golgi bodies in GnTI-GFPglyc (Figure 6A) or GALT1-GFP (Figure 6B) expressing cells, and quantified their fluorescence recovery rates (Figure 6D). For bleaching, we used the actin-depolymerizing agent latrunculin B to immobilize Golgi bodies. In both cases, Golgi bodies regained fluorescence within 7 min of the bleaching events. Fluorescence recovered to 83 and 100% of the pre-bleach value for GnTI-GFPglyc and for GALT1-GFP, respectively (Figure 6C, see Figure 6D for a summary). Thus, both fusion proteins have the same dynamic properties and cycle in and out of the Golgi stack at similar rates.

### Matrix proteins and processing enzymes strictly conform to a staged, trans-first Golgi disassembly and *cis*-first Golgi reassembly regime

**Figure 7:**
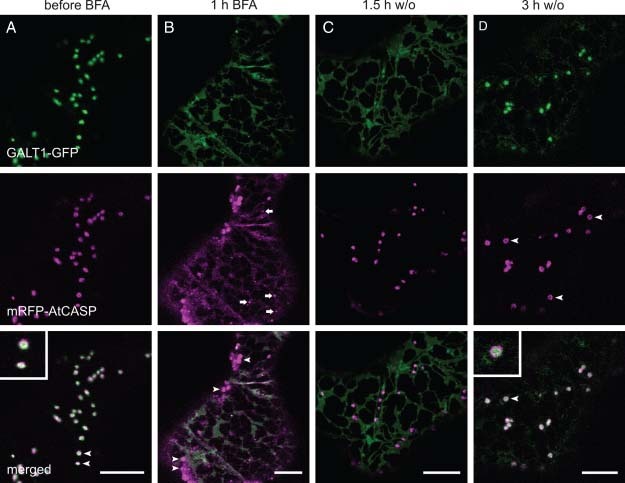
**The effects of BFA addition and washout on the *cis*/medial-Golgi matrix protein mRFP-AtCASP and the *trans*-Golgi marker GALT1-GFP.** Confocal images showing BFA treatment and washout (w/o) in wild-type tobacco leaf epidermal cells coexpressing GALT1-GFP (green) and mRFP-AtCASP (magenta). Scale bars = 10 µm. (A) Before BFA addition; (B) 1 h after BFA addition; (C) 1.5 h after BFA washout; (D) 3 h after BFA washout.

**Figure 8:**
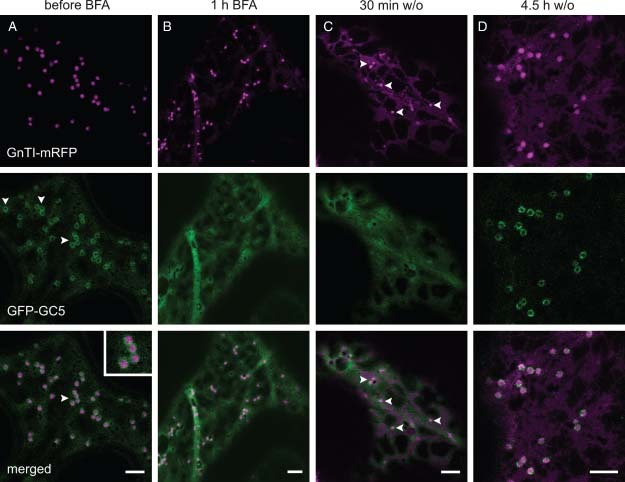
**During Golgi reconstruction the *cis*/medial-Golgi marker GnTI-mRFP recovers before the *trans*-Golgi matrix protein GFP-GC5.** Confocal images showing the BFA treatment and washout (w/o) in wild-type tobacco leaf epidermal cells coexpressing GnTI-mRFP (magenta) and GFP-GC5 (green). Scale bars = 5 µm. (A) Before BFA addition; (B) 1 h after BFA addition; (C) 30 min after BFA washout; (D) 4.5 h after BFA washout.

To gain further insight into the progressive process of Golgi disassembly and reassembly, we characterized the distributional persistence of differentially localized golgins (Table 1) in comparison to the processing enzyme reporters in response to BFA treatment and washout (Figures 7 and 8, Figure S3).

When we coexpressed the *cis/*medial-Golgi matrix protein mRFP-AtCASP together with the *trans*-Golgi enzyme GALT1-GFP, both co-located at Golgi bodies before BFA addition (Figure 7A). mRFP-AtCASP labelled a mixture of round Golgi bodies and ring structures depending on the orientation of the stacks (Figure 7A, arrowheads and inset). Those Golgi stacks that did not label rings and co-located with GALT1-GFP showed the typical shift indicating *cis*–*trans* labelling. On BFA addition, GALT1-GFP was redistributed to the ER before mRFP-AtCASP (Figure 7B), which at that time was found on a few Golgi bodies (arrowheads), in the ER and on small punctate structures (arrows). mRFP-AtCASP was never fully redistributed into the ER, but continued to label small puncta (Figure 7B, arrows), which have been shown to co-locate with the ERES marker Sar1-GTP-yellow fluorescent protein (Sar1-GTP-YFP) (30). On BFA removal, mRFP-AtCASP labelled larger punctate structures, which later co-located with GALT1-GFP at newly forming Golgi bodies (Figure 7C).

Like mRFP-AtCASP, the peripheral *trans*-Golgi matrix protein GFP-GC5 (Golgin Candidate *5*, an *Arabidopsis* homologue of the human TATA element modulatory factor) was observed as numerous fluorescent rings around Golgi stacks labelled by the *cis*/medial-Golgi enzyme GnTI-mRFP (Figure 8A, arrowheads and inset) and in addition displayed cytoplasmic labelling at steady state. Loss of GFP-GC5 from Golgi bodies and relocation to the cytoplasm upon BFA treatment preceded the redistribution of GnTI-mRFP to the ER (Figure 8B), which after drug removal reappeared before GFP-GC5, the *trans*-located matrix protein, was recruited back from the cytoplasm to Golgi membranes (Figure 8C, arrowheads indicate GnTI-mRFP labelled Golgi bodies). After 3–4.5 h, all Golgi markers tested had re-emerged from the ER or cytoplasm and re-established normal Golgi patterns (Figures 7D and 8D). The results from a BFA washout experiment with cells expressing GnTI-mRFP and AtGRIP-GFP as a peripheral marker for late-Golgi sub-compartments were similar with the exception that some GRIP-labelled puncta remained after BFA treatment which could represent TGN unaffected by the BFA treatment (Figure S3).

**Figure 9:**
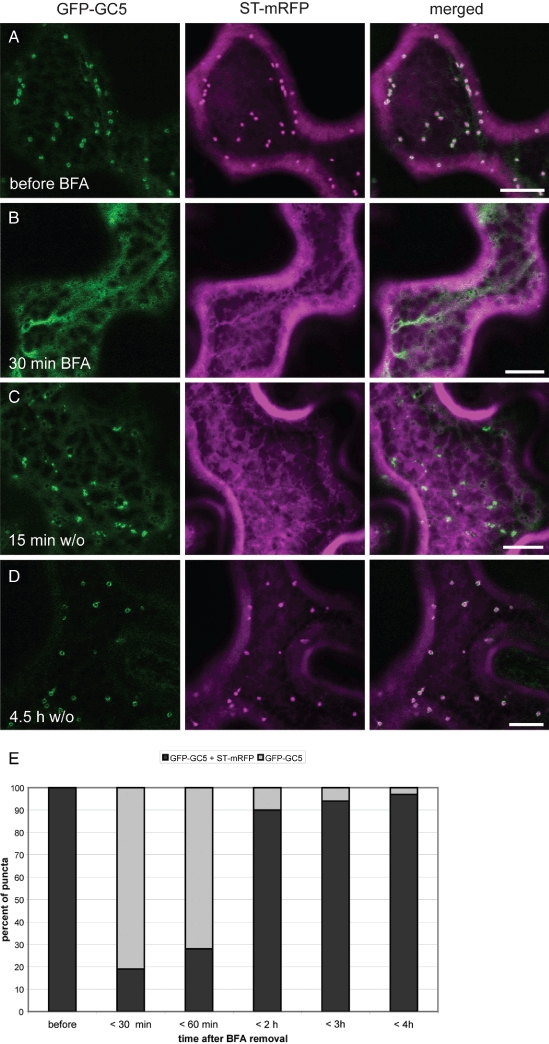
**The effects of BFA addition and washout on the *trans*-Golgi matrix protein GFP-GC5 and the *trans*-Golgi marker ST-mRFP.** Confocal images showing the BFA treatment and washout (w/o) in wild-type tobacco leaf epidermal cells coexpressing GFP-GC5 (green) and ST-mRFP (magenta). Scale bars = 10 µm. (A) Before BFA treatment; (B) 30 min after BFA addition; (C) 15 min after BFA washout; (D) 4.5 h after BFA washout. (E) Diagram showing a quantification of Golgi bodies labelled either by GFP-GC5 and ST-mRFP or solely GFP-GC5 during BFA treatment and washout.

To investigate the dynamics of the integral *cis*/medial-Golgi matrix proteins AtCASP and GC1 and the peripheral late-Golgi matrix proteins GC5 and AtGRIP relative to an established *trans*-Golgi marker, we coexpressed the GFP fusion proteins together with ST-mRFP and performed BFA washouts (Figure 9, Figures S4 and S5). GFP-GC5 and ST-mRFP both labelled Golgi bodies before BFA treatment (Figure 9A), but appeared to be redistributed to the cytoplasm or ER, respectively, at approximately the same rate (Figure 9B), indicating that they might occupy similar sub-compartments. Fifteen minutes after BFA removal GFP-GC5 labelled reforming Golgi bodies (Figure 9C) and after 4.5 h co-located with ST-mRFP (Figure 9D).

We chose this combination to quantify our results (Figure 9E), as GFP-GC5 did not label additional structures as did AtGRIP-GFP. Before BFA treatment, GFP-GC5 and ST-mRFP co-located in 100% of all puncta (*n* = 946, 24 cells). Up to 30 min after BFA removal this number had decreased to 19%, whereas 81% of puncta at this time-point contained solely GFP-GC5 (*n* = 554, 29 cells). Up to 60 min after BFA washout 28% of puncta contained both fluorescent proteins (*n* = 490, 30 cells). After 2 h 90% of punctate structures were labelled by GFP-GC5 and ST-mRFP, indicating that Golgi bodies had fully reformed (*n* = 230, 21 cells). After 3 and 4 h this number had increased to 94 and 97%, respectively (*n* = 850, 80 cells at 3 h, *n* = 1050, 57 cells at 4 h).

### A *cis*/medial-Golgi matrix protein accumulates in reforming Golgi bodies before a *cis*/medial-Golgi glycosyltransferase

**Figure 10:**
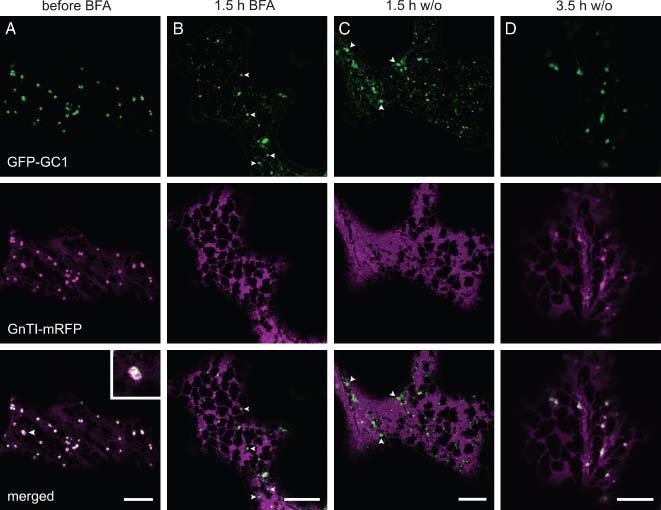
***De novo* assembly of *cis*-Golgi cisternae is preceded by structures positive for the *cis*/medial-Golgi matrix protein GFP-GC1.** Confocal images showing the BFA treatment and washout (w/o) in wild-type tobacco leaf epidermal cells coexpressing GFP-GC1 (green) and GnTI-mRFP (magenta). Scale bars = 10 µm. (A) Before BFA addition; (B) 1.5 h after BFA addition; (C) 1.5 h after BFA washout; (D) 3.5 h after BFA washout.

So far we have established that during Golgi biogenesis from the ER early-Golgi membrane markers are recruited to reforming Golgi stacks before markers for late-Golgi sub-compartments. Hence, we ultimately asked whether *de novo* assembly of *cis*-Golgi cisternae is seeded by structures positive for matrix proteins, targeted membrane proteins (i.e. processing enzymes) or a combination of both. For this purpose, we performed a BFA washout in cells expressing the *cis*/medial matrix protein GFP-GC1 together with the *cis*/medial-located GnTI-mRFP (Figure 10). Before BFA addition, GnTI-mRFP co-located with GFP-GC1 at Golgi bodies (Figure 10A), which was also detected as ring structures with GnTI-mRFP located in the centre (Figure 10A, arrowhead and inset). On BFA addition, fluorescence of both markers was lost from Golgi bodies at approximately the same time. When GnTI-mRFP had been relocated to the ER after a 1.5-h BFA incubation, GFP-GC1 labelled a few remaining Golgi bodies (Figure 10B, arrowheads), but also the cytoplasm and small punctate structures presumably representing ERES (30) (see also Figure S4). On BFA removal, GFP-GC1 quickly re-emerged from the ER and, after 1.5 h, located to larger puncta resembling Golgi bodies (Figure 10C, arrowheads), but also labelled residual cytoplasm and smaller puncta. After 2 h, GnTI-mRFP started to recover and was found to co-locate with the matrix protein at reformed Golgi bodies (data not shown). A normal Golgi pattern was re-established 3.5 h after BFA removal (Figure 10D).

## Discussion

### Sub-compartmentalization of plant *N*-glycan processing enzymes in the Golgi apparatus

*N*-glycan processing enzymes occupy successive cisternae within the Golgi stack, and by doing so, form an assembly line for the sequential processing of cargo proteins in a *cis*-to-*trans* sequence. The first evidence for their Golgi sub-compartmentalization was obtained by labelling of glycosyltransferase products using an indirect immunocytochemical approach (46–48) and immunoelectron microscopy (IEM) of, mostly, tagged plant *N*-glycan processing enzymes (13,37,49,50). So far, the successful outcome of IEM to localize the endogenous enzymes has been low because their abundance in plant cells is rather low, and antibodies specific for the native proteins that work for immunofluorescence often fail to provide good results when used for IEM. As the formation of Lewis a structures, which involves the sequential action of the transferases GALT1 and α1,4-fucosyltransferase (38), is a late-Golgi event (48), GALT1 most likely locates to the *trans*-most Golgi cisternae. Our reference marker ST was immuno-localized to the *trans*-half of Golgi stacks in leaves of *N. clevelandii*, in callus tissue and root tips of *Arabidopsis* by EM (25,50,51). Our confocal microscopy data on the intra-Golgi location of our markers are in good agreement with previous studies (37) because double-expression experiments with GnTI-mRFP, GMII-CFP and/or GALT1-GFP in tobacco leaf epidermal cells revealed subtle differences in the labelling pattern of individual Golgi bodies. This most likely reflects the targeting of the fusion proteins to distinct sets of Golgi cisternae according to their position in the *N*-glycan processing pathway.

### The fate of Golgi-resident processing enzymes and matrix proteins during Golgi disassembly

In plants, BFA and the dominant-negative GTP-locked mutant of the GTPase Sar1p (Sar1-GTP) have been successfully used to investigate the dynamics of Golgi membrane markers, matrix proteins and stack ultrastructure (4,25–28,30,36). We have used a range of both integral and peripheral Golgi-resident *N*-glycan processing enzymes and golgins as fluorescent markers for distinct intra-Golgi locations and sequentially followed their distribution during BFA treatment and inducible Sar1-GTP expression by confocal microscopy. Our multi-marker analysis showed that the first event during Golgi disassembly was the loss of *trans*-located Golgi enzymes and golgins, which was followed by a sequential relocation of medial and *cis*-Golgi enzymes into ER. *Cis*-located matrix proteins were relocated to the cytoplasm or the ER, but additionally continued to label punctate structures that might represent ERES (30). In all our experiments, the same sequence of events was observed after BFA treatment and Sar1-GTP expression, respectively.

Our biochemical investigation into the effect of BFA on cells coexpressing GnTI-mRFP and GALT1-GFP supported the confocal observations. The GALT1-GFP signal clearly shifted towards ER membrane fractions in the presence of BFA, whereas the distribution state of GnTI-mRFP remained almost unchanged. We are aware that newly synthesized protein could in theory partially contribute to the apparent ER localization of GALT1-GFP. If this was the case, pre-existing GALT1-GFP might not relocate to the ER. However, we believe that the accumulation of newly synthesized or folded protein in the ER during a 45-min BFA incubation period prior to fractionation could not contribute significantly in our biochemical analysis. Furthermore, ER localization of Golgi-located GALT1-GFP after BFA treatment is confirmed as relocation and was observed in BFA-treated cells coexpressing GnTI-mRFP and GALT1-GFP when *de novo* protein synthesis was inhibited by cycloheximide (Figure S2). In control experiments without BFA treatment, no ER localization was observed for GALT1-GFP in the presence of cycloheximide, and only little ER fluorescence was observed for GnTI-mRFP at steady state. Cycloheximide was used previously to demonstrate that BFA induces the retrograde transport of the Golgi-targeted ST-GFP into the ER in the absence of protein synthesis in tobacco leaves (28). By photobleaching experiments we excluded the possibility that a differential redistribution of the tested Golgi markers GnTI and GALT1 was due to different mobility within distinct Golgi sub-compartments. The comparative cycling rates of GnTI-GFPglyc and GALT1-GFP did not significantly differ in spite of their location to opposite sides of the Golgi stack at steady state, which is consistent with previous observations (23). We believe that these ‘resident’ Golgi enzymes are not stably associated with or physically restrained within Golgi membranes as suggested by their rapid fluorescence recovery. The differential redistribution of the early Golgi markers GnTI-mRFP and GMII-CFP (Figure 3, Figure S1) provides strong evidence against a putative hetero-oligomerization of both enzymes within Golgi cisternae as it has been demonstrated for their mammalian counterparts (52,53).

Our results are in contrast to findings in animal cells where in response to BFA Golgi membrane proteins disappeared in a *cis*-to-*trans* direction (54), although *trans*-first recycling has been postulated in a later study (55). In BFA-treated tobacco Bright Yellow 2 (BY-2) suspension cultured cells, Saint-Jore-Dupas et al. (37) observed only in some cases subtle differences in timing between early and late-Golgi markers. However, no images showing the localization of fusion proteins at different time-points of the 2-h time–course were presented. Furthermore, it was shown that BFA treatment in BY-2 cells resulted in rapid loss of coatomer followed by a subsequent loss of Golgi cisternae starting at the *cis*-face (27). Interestingly, by EM the *cis*-Golgi marker Golgi α-mannosidase I-GFP was still found in ‘*trans*-like cisternae’ before it was relocated to the ER upon prolonged BFA treatment. The authors speculated that the *cis*-Golgi marker progresses towards the *trans*-face due to cisternal maturation, but no longer can cycle back to earlier Golgi compartments in the presence of BFA. If the cisternal maturation model of Golgi transport is correct, the progression and loss of Golgi cisternae in a *cis*-to-*trans* direction are also consistent with relocation of cisternal markers in a *trans*-to-*cis* fashion.

It is noteworthy that the *cis*/medial-Golgi matrix proteins GFP-AtCASP and GFP-GC1 remained in punctate structures after complete relocation of both *trans*- and *cis*-Golgi enzyme markers to the ER. These structures have been shown to co-locate with the ERES marker Sar1-GTP-YFP (30) and our results further support the idea of a possible involvement of AtCASP and GC1 at the ERES.

### A possible alternative Golgi–ER recycling route in plants?

The observed disintegration of Golgi stacks might represent a normal retrograde recycling pathway of Golgi membrane proteins to the ER in the absence of anterograde trafficking or may reflect an abnormal process triggered by the treatments. Although it is generally accepted that COPI-coated vesicles are the agents of retrograde recycling (13,56–59), convincing evidence in favour of Golgi enzyme concentration in COPI vesicles has not yet been presented (60,61). Furthermore, BFA treatment would lead to a loss of COPI carriers in most instances. We conclude that our results are in favour of a COPI-independent retrograde pathway between the Golgi apparatus and ER. A major direct recycling pathway, maybe in the form of membrane connections, from the *trans*-Golgi cisternae to the ER would explain the initial loss of *trans*-Golgi proteins followed by earlier Golgi proteins. Such a pathway might be accentuated by BFA treatment (56) or might be COPI-independent/Rab6-dependent and therefore functional in the presence of BFA, as reported for animal cells (56,62,63). Golgi tubules as well as ER–Golgi membrane connections have long been observed in plants (5,64–67). We have observed tubules of varying length emerging from Golgi stacks after expression of fluorescent Golgi markers *in vivo* at early stages of induced Golgi stack disassembly and reassembly (Movie S1). However, we do not know whether such tubules are implicated in cargo transport or are only an expression artefact.

One other possible explanation for *trans*-first retrieval of Golgi components to the ER is a retrograde route via the *cis*-cisternae. This could be via transient connections between Golgi cisternae or via a network of tubules at the periphery of cisternae. Such a process would be the reverse of enzyme trafficking during Golgi reformation whereby *trans*-located enzymes are positioned after the *cis*-enzymes (see below). Such connectivity between cisternae of the Golgi stack has also been explored in mammalian systems (68).

### The fate of Golgi-resident processing enzymes and matrix proteins during Golgi reassembly

The capacity of the Golgi apparatus to form *de novo* from the ER after BFA washout has already been demonstrated in plant cells (23,28,36,69). In our BFA washout experiments reconstitution of Golgi stacks occurred as a staged process with a sequential re-emergence of resident Golgi processing enzymes and matrix proteins in the following order: *cis*-matrix *> cis*/ medial enzyme *> trans*-matrix *> trans* enzyme, which reflects the *cis*-to-*trans* intra-Golgi distribution of the respective Golgi proteins at steady state. A similar *cis*-to-*trans* Golgi reassembly has been reported in other systems (54,70–72). We observed that enzyme positioning in a particular Golgi sub-compartment was preceded by matrix proteins being recruited back to the same sub-compartment, an event that has been reported for mammalian cells (73). It seems unlikely that the golgin-labelled punctate structures after BFA treatment represent inert protein aggregates, as after BFA washout these structures increased in size: in the case of GFP-AtCASP from an initial diameter of 300–600 nm to that of mature Golgi stacks (30). After prolonged washout golgin labelling resembled that of normal Golgi bodies and they clearly co-located with Golgi markers, while the majority of the small punctae had disappeared. Whether matrix proteins might be involved in forming a structural platform or scaffold, which is later filled up with Golgi-resident enzymes, or whether they participate in tethering events and establishing stack polarity remains subject to speculation, but our results indicate that matrix proteins are needed at early stages of Golgi stack formation (74).

The appearance of early Golgi markers prior to late-Golgi markers is consistent with the expectations of a cisternal maturation model, in which continuous arrival of ER-derived membranes at the *cis*-side of the Golgi stack is coupled with disassembly of TGN compartments (75,76). Hypothetically, a COPII bias towards *cis*-Golgi proteins would ensure that the input to the Golgi from the ER would be enriched in early components, which may aid compartmentalization driven by maturation. In the plant field there has been more support for the cisternal maturation process for some time (66,74,77). Another explanation could be that *trans*-located enzymes can only reach *trans*-Golgi cisternae once *cis*-cisternae are present and functional.

A more detailed understanding of the fate of different Golgi-resident proteins and the plant Golgi ‘matrix’ in both molecular and dynamic terms will help to reveal whether the Golgi apparatus still constitutes a distinct organelle and hence will help to unravel the mechanistic basis of Golgi biogenesis.

## Materials and Methods

### Constructs

The following fluorescent protein fusion constructs were used in this study: pPT2M:GnTI(CTS)-mRFP (GnTI-mRFP) and p20:GnTI(CTS)-Fc-GFP (GnTI-GFPglyc) (39), p23:GMII(C_10_T)-CFP (GMII-CFP) and p20:GMII(C_10_TS)-GFP (GMII-GFP), p20:GALT1(CTS)-GFP (GALT1-GFP (38)), pVKH18-En6:ST-mRFP (ST-mRFP, (11)), pVKH18-En6:ST-GFP [ST-GFP; (28)], pMDC43:GFP-AtCASP [GFP-AtCASP; (12)], pMDC43:mRFP-AtCASP [mRFP-AtCASP; (40)], pMDC43:GFP-GC1 [GFP-GC1; (12)], pMDC83:AtGRIP-GFP [AtGRIP-GFP; (10,12)], and pMDC43:GFP-GC5 [GFP-GC5; (12)].

### Plant material and transient expression

Transient expression of fluorescent protein fusions in tobacco abaxial leaf epidermal cells was performed using the Agrobacterium-mediated infiltration technique (78). Wild-type tobacco and *N. benthamiana* plants as well as the stable tobacco mutants NII and LhGR-Sar1-GTP (30) were grown either in a greenhouse at 21°C with a 14-h light and 10-h dark regime or cultivated in a controlled growth chamber with 22°C day and night temperature, 16 h photoperiod and 50% humidity. Plants were used for infiltration after 5–6 weeks. Bacterial suspensions were infiltrated at the following optical densities (OD_600_): GnTI-mRFP 0.03, GMII-CFP 0.05, GALT1-GFP 0.08, ST-mRFP/-GFP 0.05–0.1, GFP-/mRFP-AtCASP, GFP-GC1 and GFP-GC5 0.1–0.15.

### Dexamethasone treatment

Dexamethasone treatment was performed as described by Osterrieder et al. (30). Briefly, a 20 µg/mL dexamethasone (Sigma-Aldrich, http://www.sigmaaldrich.com/) working solution with 0.02% Silwet L-77® (GE Silicones-OSi Specialties, http://www.gesilicones.com/) was freshly made and painted on the abaxial side of leaves of stable transgenic tobacco plants.

### Drug treatments

To stop Golgi stack movement in photobleaching experiments, small segments of infiltrated leaf tissue (3 × 3 mm) were treated with the actin-depolymerizing agent latrunculin B [Calbiochem, http://www.calbiochem.com/ or Sigma-Aldrich; stock solution, 1 mm in dimethyl sulphoxide (DMSO)] used at a concentration of 25 µm for 1 h (23). For ease of imaging motile organelles, leaf segments were incubated in *N*-ethylmaleimide (Sigma-Aldrich; stock solution, 1 m in DMSO) used at a concentration of 50 mm for 10 min (23,39). To block *de novo* protein synthesis, leaf segments were incubated in cycloheximide (Sigma-Aldrich; stock solution, 10 mg/mL in water) used at a concentration of 100 µg/mL for 2 h prior to BFA addition.

### BFA treatment and washout

To induce redistribution of Golgi membrane markers into the ER, infiltrated leaf segments were incubated in 100 µg/mL BFA (Sigma-Aldrich; stock solution, 10 mg/mL in DMSO) for 2–3 h. Recovery from BFA-induced Golgi disassembly was achieved by incubating treated material in water (BFA washout).

### Sampling and imaging

Sections of expressing leaves were analysed 2–4 days post-infiltration (dpi) with a Leica TCS SP2 (Leica Microsystems, http://www.leica-microsystems.com/) and a Zeiss LSM 510 or LSM 510 META (http://www.zeiss.com/) confocal laser scanning microscope. To exclude the possibility of cross-talk between fluorophores, appropriate controls were performed. Post-acquisition image processing was performed in ImageJ (http://rsbweb.nih.gov/ij/) and Adobe Photoshop CS (http://www.adobe.com/).

Imaging with Zeiss microscopes: GFP in combination with mRFP was imaged as previously described (39). For imaging CFP in combination with mRFP, the 458-nm argon laser line for CFP and the 543-nm helium/neon laser line for mRFP were used in the single-track mode. For imaging expression of GFP in combination with CFP, the 488-nm argon laser line for GFP and the 458-nm argon laser line for CFP were used alternately with line switching using the multi-track facility of the microscope. Imaging triple labelling with CFP, GFP and mRFP constructs was conducted on the Zeiss LSM 510 META. CFP and mRFP were excited simultaneously using the 458-nm argon laser line for CFP and the 543-nm helium/neon laser line for mRFP with switching after each frame to excitation of GFP with the 488-nm argon laser line.

Imaging with Leica microscope: CFP, GFP and mRFP, alone or pairwise, were imaged as described previously (39).

### Photobleaching studies

Spot photobleaching recovery measurements were performed on an upright Zeiss 510 META confocal microscope. A 63× 1.2 numerical aperture water immersion objective lens was used. Zeiss software was used to record pre-bleach and post-bleach signals and to modulate laser beam intensity. For quantification of fluorescence, signals were sampled with a wide pinhole before (five reference scans) and after bleach treatment using the 488-nm line of the argon laser set to 50% output and 1–2% transmission. Fluorescence in a region of interest (ROI) was bleached using 10 bleaching iterations of all 4 laser lines of the argon laser set to 100% transmission. Measurement of fluorescence of non-bleached areas was used to monitor and correct the occurrence of photobleaching and focus shifts during post-bleach scanning. The raw intensity data from the recovery phase were normalized by subtracting the background fluorescence and reference fluorescence from unbleached Golgi bodies labelled with ST-mRFP to compensate for laser fluctuations and focus shifts. Normalized data points were converted into a percentage scale using the following equation:

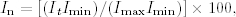
(1)
where *I*_n_ is normalized intensity, *I*_*t*_ is the intensity at any time *t*, *I*_max_ is the mean pre-bleach intensity and *I*_min_ is the immediate post-bleach intensity.

For each data set, approximately 15 Golgi bodies were photobleached. Data analysis and curve fitting were carried out using the Microsoft Excel (http://office.microsoft.com/) and GraphPad Prism 4 (http://www.graphpad.com/) software. The recovery phase of each data set was fitted with a one-phase exponential association equation:

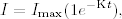
(2)
where *I* is the fluorescence intensity at time *t*, *I*_max_ is the mean pre-bleach fluorescence and *K* is the rate constant.

### Subcellular fractionation

Leaves of 6-week-old *N. benthamiana* plants were infiltrated with an agrobacterial suspension containing the constructs GnTI-mRFP and GALT1-GFP. For BFA and control treatments, 1.1 g of infiltrated leaf material each was harvested, which was either treated with a 75 µg/mL BFA working solution or treated with water containing 0.75% DMSO, which is equivalent to the DMSO concentration in the BFA working solution, for 45 min each. To separate cell organelles from crude cellular extracts, liquid was drained off and the treated leaf material was resuspended in 10 mL pre-chilled extraction buffer [100 mm 2-amino-2-(hydroxymethyl)-1,3-propandediol (TRIS)–HCl pH 7.5, 1 mm dithioerythritol (DTE), 3 mm magnesium chloride (MgCl_2_), 0.1 mm EDTA, 1 mm phenylmethylsulphonyl fluoride (PMSF), 250 mm sucrose] using an Ultra-Turrax homogenisator with three 20-second bursts. The slurry was centrifuged at 3000×***g*** for 5 min at 4°C to remove nuclei, mitochondria and undisrupted cells and the supernatant was filtered. After pre-centrifugation of the filtrate at 10 000 ×***g*** for 10 min at 4°C, the supernatant was ultracentrifuged at 100 000 ×***g*** for 40 min at 4°C to obtain a microsomal pellet. The pellets were resuspended in 500 µL extraction buffer, loaded onto a discontinuous sucrose density gradient (20–52%) and ultracentrifuged in an SW 41 Ti swing-out rotor (Beckman,http://www.beckmancoulter.com/) at 100 000 ×***g*** for 5 h at 4°C. Twenty-one 0.6-mL fractions were collected from the top of the gradient and protein in sucrose fractions was precipitated using the chloroform/methanol procedure (79). Equal volumes of each fraction were separated on SDS–PAGE gels, silver-stained and transferred onto nitrocellulose membranes (GE Healthcare, http://www4.gelifesciences.com/). Proteins were probed with the following antibodies: mouse anti-GFP (Roche, http://www.roche-applied-science.com/), rabbit anti-mRFP (US Biological, http://www.usbio.net/) or rabbit anti-BiP. For reprobing with a different antibody, the nitrocellulose membrane was incubated for 60 min at 65°C in stripping buffer [0.1 m glycine pH 2.0, 0.5 m sodium chloride (NaCl), 1% SDS] followed by six 10-min washes at room temperature using 1× phosphate-buffered saline (PBS) plus 1% Tween®-20 (Sigma-Aldrich). IImageJ was used to quantify the relative intensities of western blot bands by following a tutorial written by Luke Miller (http://www.lukemiller.org/journal/journal.html).

## Supporting Information

**Figure S1: Redistribution of Golgi-resident markers during Golgi disassembly.** GMII-CFP is redistributed into the ER before GnTI-mRFP (A), and GALT1-GFP is redistributed into the ER before GMII-CFP (B) during Golgi disassembly. (A) Time series showing GnTI-mRFP (magenta) and GMII-CFP (green) either transiently coexpressed in leaves of wild-type tobacco plants treated with BFA (100 µg/mL) for 1.5 and 2 h or in leaves of stable Sar1-GTP-inducible tobacco plants treated with dexamethasone (20 µg/mL) for 20 or 24 h. Arrowheads indicate Golgi bodies labelled by GnTI-mRFP solely. (B) Time series showing GMII-CFP (magenta) and GALT1-GFP (green) either transiently coexpressed in leaves of wild-type tobacco plants treated with BFA (100 µg/mL) for 1.5 and 2 h or in leaves of stable Sar1-GTP-inducible tobacco plants treated with dexamethasone (20 µg/mL) for 19 and 23 h. Arrowheads indicate Golgi bodies labelled by GMII-CFP solely. Double-colour images (merge of green and magenta channels) were obtained by confocal microscopy at different time-points of each experiment. Scale bars = 10 µm.

**Figure S2: BFA induces the sequential redistribution of GnTI-mRFP and ST-GFP into the ER in the presence of a protein synthesis inhibitor.** Confocal images showing BFA treatment of wild-type tobacco leaf epidermal cells coexpressing GnTI-mRFP (magenta) and ST-GFP (green) in the presence of cycloheximide (CHX, 100 µg/mL). Cells were treated with CHX for 2 h before BFA was added. Scale bars = 10 µm. (A) One hour after CHX addition, GnTI-mRFP and ST-GFP co-located at Golgi bodies. (B) Forty-five minutes after BFA addition, both constructs have partially been relocated to the ER in the absence of *de novo* protein synthesis, but at the same time still labelled a couple of remaining Golgi stacks. The merged image of both channels shows that some stacks were labelled only by GnTI-mRFP at that stage (arrowheads).

**Figure S3: BFA washout in cells coexpressing the *cis*/medial-Golgi marker GnTI-mRFP and the *trans*-Golgi/TGN-located matrix protein AtGRIP-GFP.** Confocal images showing the BFA treatment and washout (w/o) in wild-type tobacco leaf epidermal cells coexpressing GnTI-mRFP (magenta) and AtGRIP-GFP (green). Scale bars = 5 µm. (A) Before BFA addition, GnTI-mRFP and AtGRIP-GFP co-located at Golgi bodies. Inset: high magnification of Golgi stacks (arrowheads in merged image) where AtGRIP-GFP was observed as fluorescent rings around the central GnTI-mRFP signal. (B) Thirty minutes after BFA addition (100 µg/mL), AtGRIP-GFP had been completely relocated into the cytoplasm, whereas GnTI-mRFP still concentrated at Golgi bodies. Note that AtGRIP-GFP also labelled additional puncta, which might represent the TGN or post-Golgi compartments (arrows). (C) Three and a half hours after BFA washout, punctate structures, more of them labelled by GnTI-mRFP, increased in size. (D) Four and a half hours after BFA washout, the Golgi apparatus had reformed and both GnTI-mRFP and AtGRIP-GFP co-located at Golgi bodies.

**Figure S4: BFA washout in cells coexpressing *cis*/medial-Golgi matrix proteins and the *trans*-Golgi marker ST-mRFP.** Confocal images showing the BFA treatment and washout (w/o) in wild-type tobacco leaf epidermal cells coexpressing GFP-AtCASP (green, A–D) or GFP-GC1 (green, E–H) and ST-mRFP (magenta, A–H). Scale bars = 10 µm. (A) Before BFA addition both markers co-located at the Golgi apparatus. (B) After 75 min of BFA treatment, GFP-AtCASP and ST-mRFP have been redistributed into the ER. GFP-AtCASP labelled additional small puncta (arrowheads). (C) Fifteen minutes after BFA washout punctate structures labelled solely by GFP-AtCASP increased in size (arrowheads). (D) Three hours after BFA washout the Golgi apparatus had reformed and both GFP-AtCASP and ST-mRFP co-located at Golgi bodies. (E) Before BFA addition GFP-GC1 and ST-mRFP co-located at the Golgi apparatus. The increased ER labelling of ST-mRFP is most likely an effect of over-expression of the golgin and is often observed. (F) After 2 h of BFA treatment, ST-mRFP had been redistributed into the ER. GFP-GC1 labelled the cytoplasm and additional puncta of varying sizes (arrowheads). (G) One and a half hours after BFA washout, punctate structures labelled solely by GFP-GC1 increased in size (arrowheads). (H) Four and a half hours after BFA washout, GFP-GC1 and ST-mRFP co-located at the Golgi bodies (arrowheads).

**Figure S5: BFA washout in cells coexpressing the *trans*-Golgi/TGN-located Golgi matrix protein AtGRIP-GFP and the *trans*-Golgi marker ST-mRFP.** Confocal images showing the BFA treatment and washout (w/o) in wild-type tobacco leaf epidermal cells coexpressing AtGRIP-GFP (green) and ST-mRFP (magenta). Scale bars = 10 µm. (A) Before BFA addition. (B) After 1 h of BFA treatment, AtGRIP-GFP dissociated off Golgi bodies labelled by ST-mRFP (arrowheads). (C) In this cell, 1 h after BFA treatment, ST-mRFP had been redistributed into the ER, whereas AtGRIP-GFP labelled the cytoplasm and puncta presumably representing the TGN. (D) Thirty minutes after BFA washout puncta labelled solely by AtGRIP-GFP increased in number and size (arrowheads). (E) Both markers co-located at reformed Golgi bodies 4 h after BFA washout.

**Movie S1: Tubules extending from Golgi bodies are regularly observed during Golgi disassembly and reformation.** Time-lapse analysis showing movement of Golgi-associated tubules at 4 h after BFA washout in tobacco leaf epidermal cells expressing GFP-AtCASP.

## References

[1] Hawes C, Osterrieder A, Hummel E, Sparkes I (2008). The plant ER-Golgi interface.. Traffic.

[2] Faso C, Boulaflous A, Brandizzi F (2009). The plant Golgi apparatus: last 10 years of answered and open questions.. FEBS Lett.

[3] Robinson D, Langhans M, Saint-Jore-Dupas C, Hawes C (2008). BFA effects are tissue and not just plant specific.. Trends Plant Sci.

[4] daSilva L, Snapp E, Denecke J, Lippincott-Schwartz J, Hawes C, Brandizzi F (2004). Endoplasmic reticulum export sites and Golgi bodies behave as single mobile secretory units in plant cells.. Plant Cell.

[5] Sparkes I, Ketelaar T, de Ruijter N, Hawes C (2009). Grab a Golgi: laser trapping of Golgi bodies reveals in vivo interactions with the endoplasmic reticulum.. Traffic.

[6] Short B, Haas A, Barr F (2005). Golgins and GTPases, giving identity and structure to the Golgi apparatus.. Biochim Biophys Acta.

[7] Lupashin V, Sztul E (2005). Golgi tethering factors.. Biochim Biophys Acta.

[8] Sztul E, Lupashin V (2009). Role of vesicle tethering factors in the ER-Golgi membrane traffic.. FEBS Lett.

[9] Latijnhouwers M, Hawes C, Carvalho C (2005). Holding it all together? Candidate proteins for the plant Golgi matrix.. Curr Opin Plant Biol.

[10] Latijnhouwers M, Hawes C, Carvalho C, Oparka K, Gillingham A, Boevink P (2005). An *Arabidopsis* GRIP domain protein locates to the trans-Golgi and binds the small GTPase ARL1.. Plant J.

[11] Renna L, Hanton S, Stefano G, Bortolotti L, Misra V, Brandizzi F (2005). Identification and characterization of AtCASP, a plant transmembrane Golgi matrix protein.. Plant Mol Biol.

[12] Latijnhouwers M, Gillespie T, Boevink P, Kriechbaumer V, Hawes C, Carvalho C (2007). Localization and domain characterization of *Arabidopsis* golgin candidates.. J Exp Bot.

[13] Staehelin L, Kang B (2008). Nanoscale architecture of endoplasmic reticulum export sites and of Golgi membranes as determined by electron tomography.. Plant Physiol.

[14] Saint-Jore-Dupas C, Gomord V, Paris N (2004). Protein localization in the plant Golgi apparatus and the trans-Golgi network.. Cell Mol Life Sci.

[15] Pfeffer S (2007). Unsolved mysteries in membrane traffic.. Annu Rev Biochem.

[16] Tu L, Banfield D (2010). Localization of Golgi-resident glycosyltransferases.. Cell Mol Life Sci.

[17] Lippincott-Schwartz J, Roberts T, Hirschberg K (2000). Secretory protein trafficking and organelle dynamics in living cells.. Annu Rev Cell Dev Biol.

[18] Ward T, Brandizzi F (2004). Dynamics of proteins in Golgi membranes: comparisons between mammalian and plant cells highlighted by photobleaching techniques.. Cell Mol Life Sci.

[19] Storrie B (2005). Maintenance of Golgi apparatus structure in the face of continuous protein recycling to the endoplasmic reticulum: making ends meet.. Int Rev Cytol.

[20] Nichols B, Pelham H (1998). SNAREs and membrane fusion in the Golgi apparatus.. Biochim Biophys Acta.

[21] Zaal K, Smith C, Polishchuk R, Altan N, Cole N, Ellenberg J, Hirschberg K, Presley J, Roberts T, Siggia E, Phair R, Lippincott-Schwartz J (1999). Golgi membranes are absorbed into and reemerge from the ER during mitosis.. Cell.

[22] Miles S, McManus H, Forsten K, Storrie B (2001). Evidence that the entire Golgi apparatus cycles in interphase HeLa cells: sensitivity of Golgi matrix proteins to an ER exit block.. J Cell Biol.

[23] Brandizzi F, Snapp E, Roberts A, Lippincott-Schwartz J, Hawes C (2002). Membrane protein transport between the endoplasmic reticulum and the Golgi in tobacco leaves is energy dependent but cytoskeleton independent: evidence from selective photobleaching.. Plant Cell.

[24] Chardin P, McCormick F (1999). Brefeldin A: the advantage of being uncompetitive.. Cell.

[25] Boevink P, Oparka K, Santa Cruz S, Martin B, Betteridge A, Hawes C (1998). Stacks on tracks: the plant Golgi apparatus traffics on an actin/ER network.. Plant J.

[26] Takeuchi M, Ueda T, Sato K, Abe H, Nagata T, Nakano A (2000). A dominant negative mutant of sar1 GTPase inhibits protein transport from the endoplasmic reticulum to the Golgi apparatus in tobacco and *Arabidopsis* cultured cells.. Plant J.

[27] Ritzenthaler C, Nebenführ A, Movafeghi A, Stussi-Garaud C, Behnia L, Pimpl P, Staehelin L, Robinson D (2002). Reevaluation of the effects of brefeldin A on plant cells using tobacco Bright Yellow 2 cells expressing Golgi-targeted green fluorescent protein and COPI antisera.. Plant Cell.

[28] Saint-Jore C, Evins J, Batoko H, Brandizzi F, Moore I, Hawes C (2002). Redistribution of membrane proteins between the Golgi apparatus and endoplasmic reticulum in plants is reversible and not dependent on cytoskeletal networks.. Plant J.

[29] Lee M, Min M, Lee Y, Jin J, Shin D, Kim D, Lee K, Hwang I (2002). ADP-ribosylation factor 1 of *Arabidopsis* plays a critical role in intracellular trafficking and maintenance of endoplasmic reticulum morphology in *Arabidopsis*. Plant Physiol.

[30] Osterrieder A, Hummel E, Carvalho C, Hawes C (2010). Golgi membrane dynamics after induction of a dominant-negative mutant Sar1 GTPase in tobacco.. J Exp Bot.

[31] Lippincott-Schwartz J, Yuan L, Bonifacino J, Klausner R (1989). Rapid redistribution of Golgi proteins into the ER in cells treated with brefeldin A: evidence for membrane cycling from Golgi to ER.. Cell.

[32] Doms R, Russ G, Yewdell J (1989). Brefeldin A redistributes resident and itinerant Golgi proteins to the endoplasmic reticulum.. J Cell Biol.

[33] Seemann J, Jokitalo E, Pypaert M, Warren G (2000). Matrix proteins can generate the higher order architecture of the Golgi apparatus.. Nature.

[34] Ward T, Polishchuk R, Caplan S, Hirschberg K, Lippincott-Schwartz J (2001). Maintenance of Golgi structure and function depends on the integrity of ER export.. J Cell Biol.

[35] Yoshimura S, Yamamoto A, Misumi Y, Sohda M, Barr F, Fujii G, Shakoori A, Ohno H, Mihara K, Nakamura N (2004). Dynamics of Golgi matrix proteins after the blockage of ER to Golgi transport.. J Biochem.

[36] Langhans M, Hawes C, Hillmer S, Hummel E, Robinson D (2007). Golgi regeneration after brefeldin A treatment in BY-2 cells entails stack enlargement and cisternal growth followed by division.. Plant Physiol.

[37] Saint-Jore-Dupas C, Nebenführ A, Boulaflous A, Follet-Gueye M, Plasson C, Hawes C, Driouich A, Faye L, Gomord V (2006). Plant N-glycan processing enzymes employ different targeting mechanisms for their spatial arrangement along the secretory pathway.. Plant Cell.

[38] Strasser R, Bondili J, Vavra U, Schoberer J, Svoboda B, Glössl J, Léonard R, Stadlmann J, Altmann F, Steinkellner H, Mach L (2007). A unique beta1,3-galactosyltransferase is indispensable for the biosynthesis of N-glycans containing Lewis a structures in *Arabidopsis thaliana*. Plant Cell.

[39] Schoberer J, Vavra U, Stadlmann J, Hawes C, Mach L, Steinkellner H, Strasser R (2009). Arginine/lysine residues in the cytoplasmic tail promote ER export of plant glycosylation enzymes.. Traffic.

[40] Osterrieder A, Carvalho C, Latijnhouwers M, Johansen J, Stubbs C, Botchway S, Hawes C (2009). Fluorescence lifetime imaging of interactions between Golgi tethering factors and small GTPases in plants.. Traffic.

[41] Denecke J, Goldman M, Demolder J, Seurinck J, Botterman J (1991). The tobacco luminal binding protein is encoded by a multigene family.. Plant Cell.

[42] Chen Y, Randlett M, Findell J, Schaller G (2002). Localization of the ethylene receptor ETR1 to the endoplasmic reticulum of *Arabidopsis*. J Biol Chem.

[43] Tamura K, Shimada T, Kondo M, Nishimura M, Hara-Nishimura I (2005). KATAMARI1/MURUS3 Is a novel Golgi membrane protein that is required for endomembrane organization in *Arabidopsis*. Plant Cell.

[44] Ueda H, Yokota E, Kutsuna N, Shimada T, Tamura K, Shimmen T, Hasezawa S, Dolja V, Hara-Nishimura I (2010). Myosin-dependent endoplasmic reticulum motility and F-actin organization in plant cells.. Proc Natl Acad Sci U S A.

[45] Dunkley T, Hester S, Shadforth I, Runions J, Weimar T, Hanton S, Griffin J, Bessant C, Brandizzi F, Hawes C, Watson R, Dupree P, Lilley K (2006). Mapping the Arabidopsis organelle proteome.. Proc Natl Acad Sci U S A.

[46] Lainé A, Gomord V, Faye L (1991). Xylose-specific antibodies as markers of subcompartmentation of terminal glycosylation in the Golgi apparatus of sycamore cells.. FEBS Lett.

[47] Fitchette-Lain A-C, Gomord V, Chekkafi A, Faye L (1994). Distribution of xylosylation and fucosylation in the plant Golgi apparatus.. Plant J.

[48] Fitchette A, Cabanes-Macheteau M, Marvin L, Martin B, Satiat-Jeunemaitre B, Gomord V, Crooks K, Lerouge P, Faye L, Hawes C (1999). Biosynthesis and immunolocalization of Lewis a-containing N-glycans in the plant cell.. Plant Physiol.

[49] Nebenführ A, Gallagher L, Dunahay T, Frohlick J, Mazurkiewicz A, Meehl J, Staehelin L (1999). Stop-and-go movements of plant Golgi stacks are mediated by the acto-myosin system.. Plant Physiol.

[50] Reichardt I, Stierhof Y, Mayer U, Richter S, Schwarz H, Schumacher K, Jürgens G (2007). Plant cytokinesis requires de novo secretory trafficking but not endocytosis.. Curr Biol.

[51] Wee E, Sherrier D, Prime T, Dupree P (1998). Targeting of active sialyltransferase to the plant Golgi apparatus.. Plant Cell.

[52] Nilsson T, Hoe M, Slusarewicz P, Rabouille C, Watson R, Hunte F, Watzele G, Berger E, Warren G (1994). Kin recognition between medial Golgi enzymes in HeLa cells.. EMBO J.

[53] Nilsson T, Rabouille C, Hui N, Watson R, Warren G (1996). The role of the membrane-spanning domain and stalk region of N-acetylglucosaminyltransferase I in retention, kin recognition and structural maintenance of the Golgi apparatus in HeLa cells.. J Cell Sci.

[54] Alcalde J, Bonay P, Roa A, Vilaro S, Sandoval I (1992). Assembly and disassembly of the Golgi complex: two processes arranged in a cis-trans direction.. J Cell Biol.

[55] Yang W, Storrie B (1998). Scattered Golgi elements during microtubule disruption are initially enriched in trans-Golgi proteins.. Mol Biol Cell.

[56] Storrie B, Pepperkok R, Nilsson T (2000). Breaking the COPI monopoly on Golgi recycling.. Trends Cell Biol.

[57] Pimpl P, Movafeghi A, Coughlan S, Denecke J, Hillmer S, Robinson D (2000). In situ localization and in vitro induction of plant COPI-coated vesicles.. Plant Cell.

[58] Lanoix J, Ouwendijk J, Stark A, Szafer E, Cassel D, Dejgaard K, Weiss M, Nilsson T (2001). Sorting of Golgi resident proteins into different subpopulations of COPI vesicles: a role for ArfGAP1.. J Cell Biol.

[59] Martinez-Menárguez J, Prekeris R, Oorschot V, Scheller R, Slot J, Geuze H, Klumperman J (2001). Peri-Golgi vesicles contain retrograde but not anterograde proteins consistent with the cisternal progression model of intra-Golgi transport.. J Cell Biol.

[60] Polishchuk R, Mironov A (2004). Structural aspects of Golgi function.. Cell Mol Life Sci.

[61] Kweon H, Beznoussenko G, Micaroni M, Polishchuk R, Trucco A, Martella O, Di Giandomenico D, Marra P, Fusella A, Di Pentima A, Berger E, Geerts W, Koster A, Burger K, Luini A (2004). Golgi enzymes are enriched in perforated zones of golgi cisternae but are depleted in COPI vesicles.. Mol Biol Cell.

[62] Girod A, Storrie B, Simpson J, Johannes L, Goud B, Roberts L, Lord J, Nilsson T, Pepperkok R (1999). Evidence for a COP-I-independent transport route from the Golgi complex to the endoplasmic reticulum.. Nat Cell Biol.

[63] White J, Johannes L, Mallard F, Girod A, Grill S, Reinsch S, Keller P, Tzschaschel B, Echard A, Goud B, Stelzer E (1999). Rab6 coordinates a novel Golgi to ER retrograde transport pathway in live cells.. J Cell Biol.

[64] Kristen U (1980). Endoplasmic reticulum-dictyosome interconnections in ligula cells of Isoetes lacustris.. Eur J Cell Biol.

[65] Harris N, Oparka KJ (1983). Connections between dictyosomes, ER and GERL in cotyledons of mung bean (*Vigna radiata* L.).. Protoplasma.

[66] Hawes C, Satiat-Jeunemaitre B (2005). The plant Golgi apparatus–going with the flow.. Biochim Biophys Acta.

[67] Moreau P, Brandizzi F, Hanton S, Chatre L, Melser S, Hawes C, Satiat-Jeunemaitre B (2007). The plant ER-Golgi interface: a highly structured and dynamic membrane complex.. J Exp Bot.

[68] Patterson G, Hirschberg K, Polishchuk R, Gerlich D, Phair R, Lippincott-Schwartz J (2008). Transport through the Golgi apparatus by rapid partitioning within a two-phase membrane system.. Cell.

[69] Satiat-Jeunemaître B, Hawes C (1992). Redistribution of a Golgi glycoprotein in plant cells treated with Brefeldin A.. J Cell Sci.

[70] Puri S, Linstedt A (2003). Capacity of the golgi apparatus for biogenesis from the endoplasmic reticulum.. Mol Biol Cell.

[71] Ho H, He C, de Graffenried C, Murrells L, Warren G (2006). Ordered assembly of the duplicating Golgi in Trypanosoma brucei.. Proc Natl Acad Sci U S A.

[72] Hummel E, Schmickl R, Hinz G, Hillmer S, Robinson D (2007). Brefeldin A action and recovery in Chlamydomonas are rapid and involve fusion and fission of Golgi cisternae.. Plant Biol (Stuttg).

[73] Jiang S, Rhee S, Gleeson P, Storrie B (2006). Capacity of the Golgi apparatus for cargo transport prior to complete assembly.. Mol Biol Cell.

[74] Hawes C, Schoberer J, Hummel E, Osterrieder A (2010). Biogenesis of the plant Golgi apparatus.. Biochem Soc Trans.

[75] Bannykh S, Balch W (1997). Membrane dynamics at the endoplasmic reticulum-Golgi interface.. J Cell Biol.

[76] Glick B, Elston T, Oster G (1997). A cisternal maturation mechanism can explain the asymmetry of the Golgi stack.. FEBS Lett.

[77] Beams H, Kessel R (1968). The Golgi apparatus: structure and function.. Int Rev Cytol.

[78] Sparkes I, Runions J, Kearns A, Hawes C (2006). Rapid, transient expression of fluorescent fusion proteins in tobacco plants and generation of stably transformed plants.. Nat Protoc.

[79] Wessel D, Flügge U (1984). A method for the quantitative recovery of protein in dilute solution in the presence of detergents and lipids.. Anal Biochem.

