# Supplementary figures and images for: Sequential Depletion and Acquisition of Proteins during Golgi Stack Disassembly and Reformation
Source: Traffic. 2010 Aug 18;11(11):1429–44. doi: 10.1111/j.1600-0854.2010.01106.x (PMC3039244; doi:10.1111/j.1600-0854.2010.01106.x)

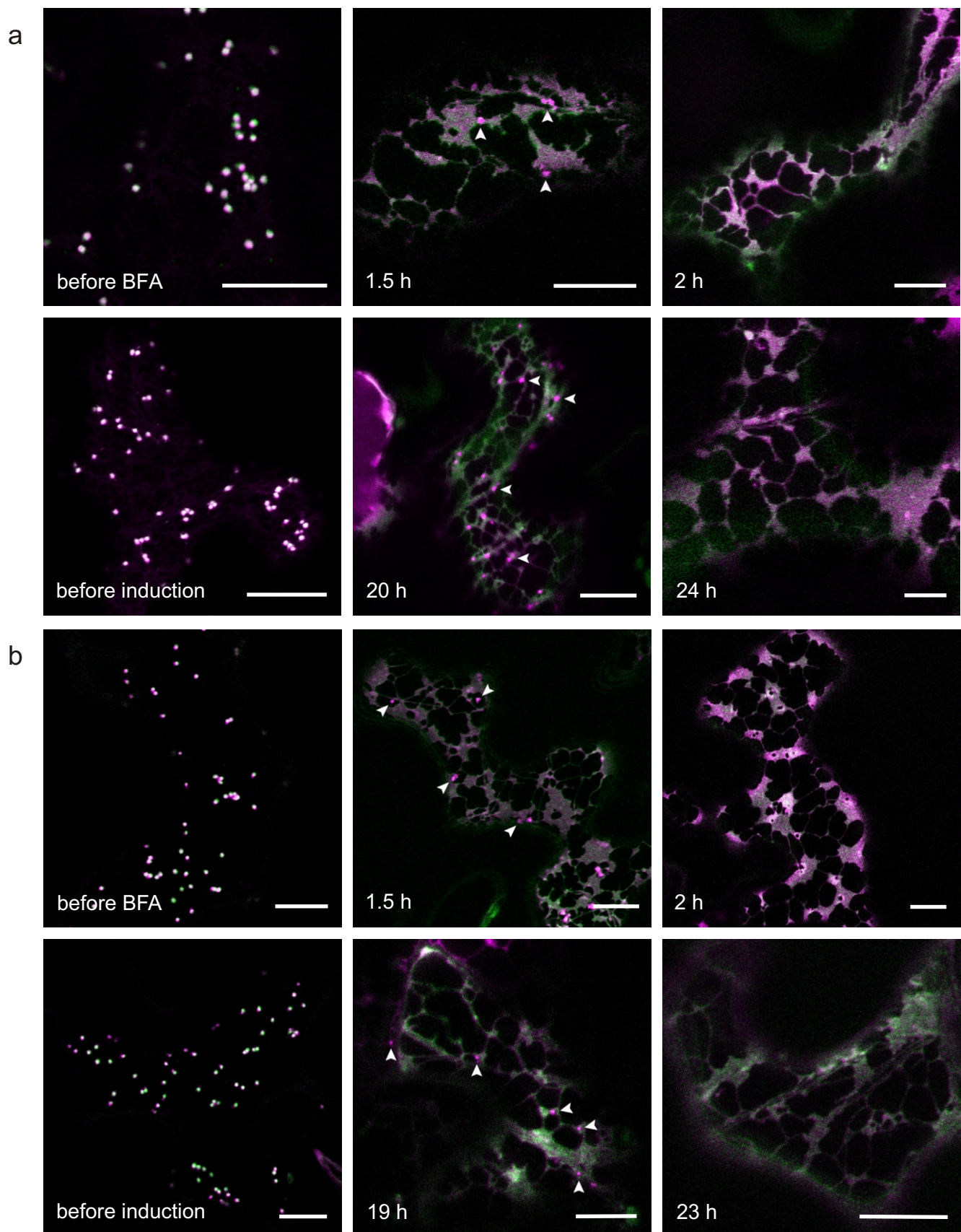

Figure S1

Supplement: Supplementary file 1 [file tra0011-1429-SD1.pdf]

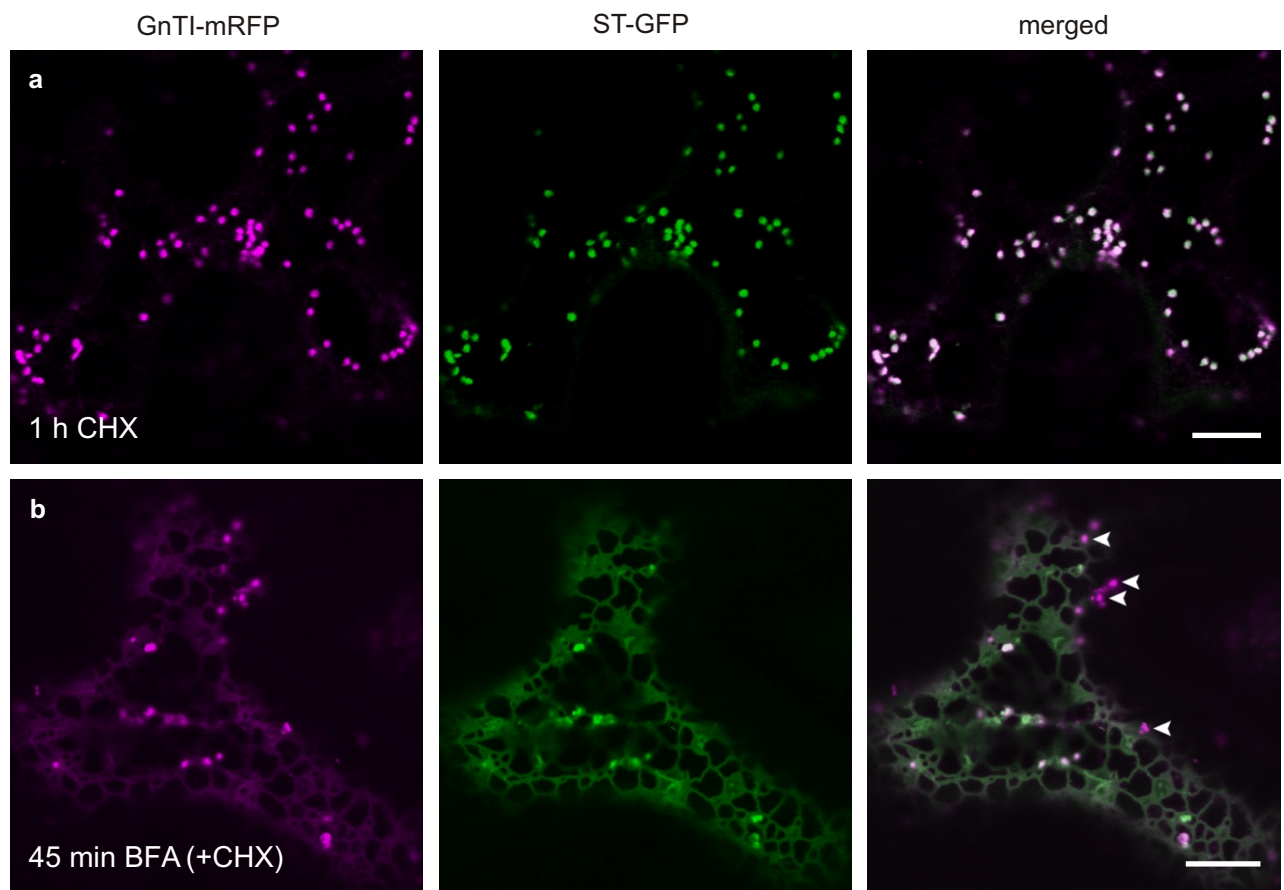

Figure S2

Supplement: Supplementary file 2 [file tra0011-1429-SD2.pdf]

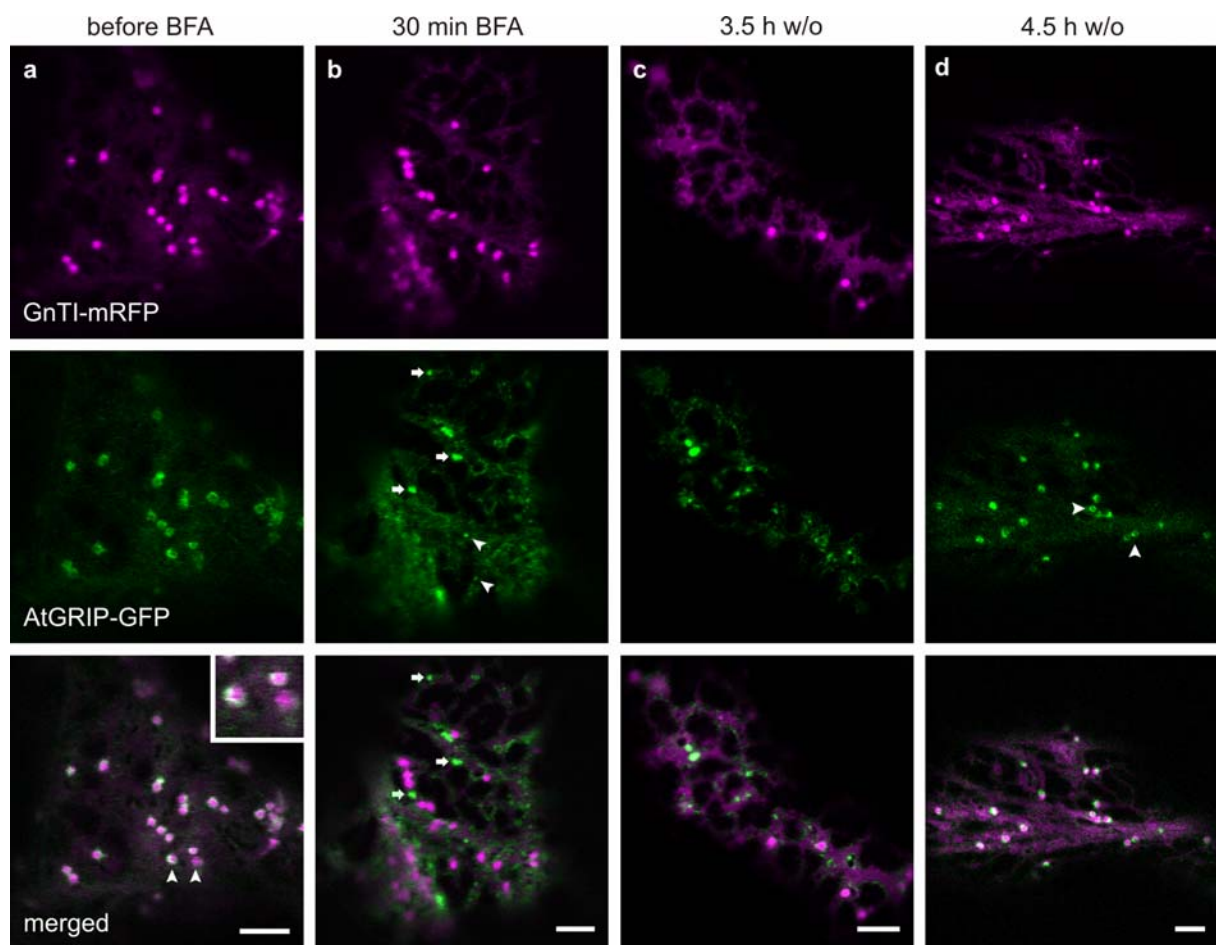

Figure S3

Supplement: Supplementary file 3 [file tra0011-1429-SD3.pdf]

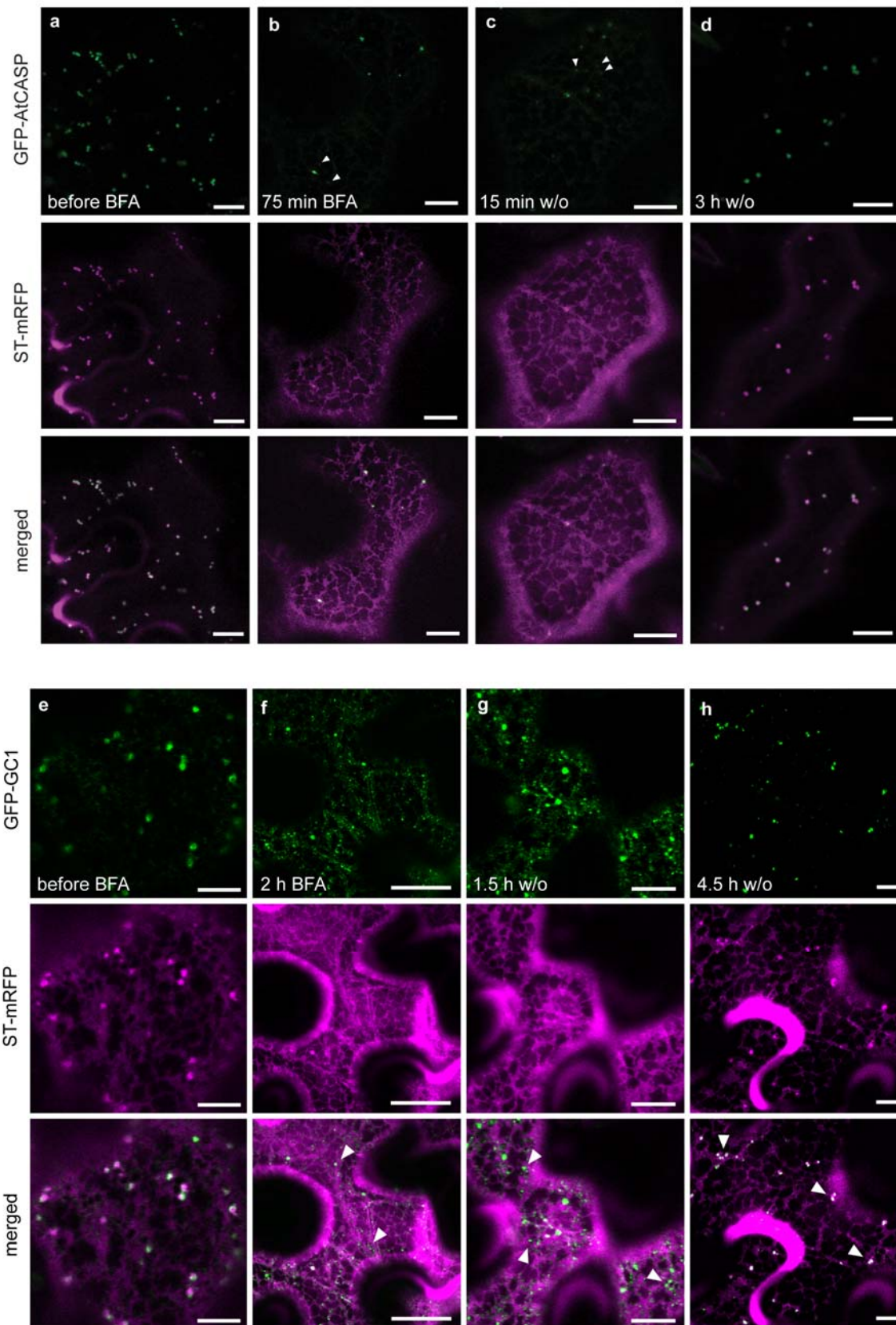

Figure S4

Supplement: Supplementary file 4 [file tra0011-1429-SD4.pdf]

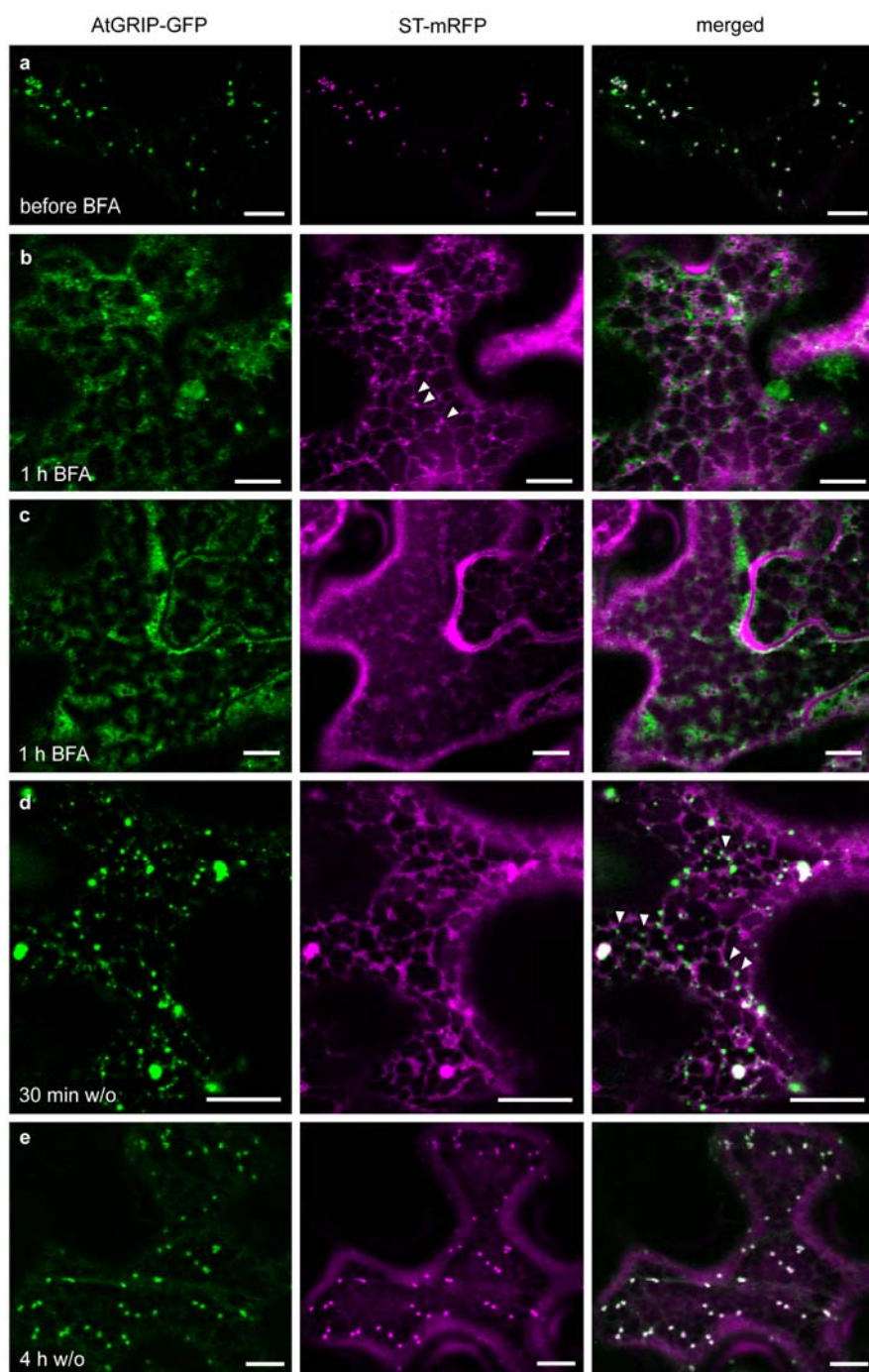

Figure S5

Supplement: Supplementary file 5 [file tra0011-1429-SD5.pdf]
